# Inactivation of Scrapie Prions by the Electrically Charged Disinfectant CAC-717

**DOI:** 10.3390/pathogens9070536

**Published:** 2020-07-03

**Authors:** Akikazu Sakudo, Yoshifumi Iwamaru, Koichi Furusaki, Makoto Haritani, Rumiko Onishi, Morikazu Imamura, Takashi Yokoyama, Yasuhiro Yoshikawa, Takashi Onodera

**Affiliations:** 1 School of Veterinary Medicine, Okayama University of Science, Imabari, Ehime 794-8555, Japan; a-sakudo@vet.ous.ac.jp (A.S.); y-yoshikawa@vet.ous.ac.jp (Y.Y.); 2 National Institute of Animal Health, Ibaraki 305-1002, Japan; gan@affrc.go.jp (Y.I.); ayokoyam@g.ecc.u-tokyo.ac.jp (T.Y.); 3 Mineral Activation Research Institute, Kumamoto 865-0023, Japan; furusaki@ind.bbiq.jp; 4 Research Center for Food Safety, University of Tokyo, Tokyo 113-8657, Japan; aharitani@mail.ecc.u-tokyo.ac.jp; 5 Santa Mineral Co., Ltd., Minato-ku, Tokyo 105-0013, Japan; rumiko@santa-mineral.co.jp; 6 Faculty of Medicine, University of Miyazaki, Kiyotake-cho, Miyazaki 889-1692, Japan; morikazu_imamura@med.miyazaki-u.ac.jp

**Keywords:** anti-prion compound, calcium hydrogen carbonate, mesoscopic structure, scrapie prions

## Abstract

Previous studies have revealed that the electrically charged disinfectant CAC-717 has strong virucidal and bactericidal effects but is safe for humans and animals. In this study, CAC-717 was further evaluated for its potential effects as a disinfectant against scrapie prions. Western blotting showed that CAC-717 reduced the amount of the abnormal isoform of prion protein (PrP^Sc^) in prion-infected cell (ScN2a) lysates. Furthermore, the reduction of prion transmissibility was confirmed by a mouse bioassay, in which mice injected with scrapie prions pre-treated with CAC-717 survived longer than those injected with untreated scrapie prions. Lastly, to evaluate the seeding activity of ScN2a cell lysates treated with CAC-717, quantitative protein misfolding cyclic amplification (PMCA) was performed directly on ScN2a cell lysates treated with CAC-717, which showed that the median dose of PMCA (PMCA_50_) dropped from log9.95 to log5.20 after CAC-717 treatment, indicating more than a 4 log reduction. This suggests that the seeding activity of PrP^Sc^ is decreased by CAC-717. Collectively, these results suggest that CAC-717 has anti-prion activity, reducing both PrP^Sc^ conversion activity and prion transmissibility; thus, CAC-717 will be useful as a novel disinfectant in prion diseases.

## 1. Introduction

Prion diseases, also known as transmissible spongiform encephalopathies, are a group of fatal neurodegenerative disorders that affect humans and animals [1,2]. They develop through deposition of the abnormal isoform of prion protein (PrP^Sc^) in the central nervous system. Conformational change of cellular prion protein (PrP^C^) generates PrP^Sc^, which constitutes prion transmissibility. PrP^Sc^ shows increased β-sheet content and proteinase-K (PK) resistance and is highly resistant to chemical and physical treatments [3].

Cases of iatrogenic Creutzfeldt-Jakob disease (iCJD) caused by iatrogenic transmission of abnormal prion protein highlight the need to inactivate prion proteins and prevent transmissibility from medical apparatus. However, neither conventional sterilization procedures nor exposure to ultraviolet or γ-ray irradiation successfully inactivates prions [4,5].

While extreme conditions are recommended for inactivation of prions by autoclaving (134 °C, 18 min) [6], transmissibility of some prions has been shown to remain even after dry-heating at temperatures of 400 °C [7]. In addition, treatment with sodium dodecyl sulfate (SDS), sodium hydroxide (NaOH), and sodium hypochlorite is known to inactivate prions [8,9,10], but these chemicals are generally impractical because of their corrosiveness [8]. Although alkaline detergents and enzyme detergents may reduce prion infectivity on medical devices [4,11,12,13,14], some enzymatic detergents have been found to enhance the resistance of prions to autoclaving [15,16]. Thus, care must be taken in the combinatorial use of these approaches. Other disinfectants such as Environ LpH have proven to be effective for inactivating scrapie prion, but it remains unclear which components or combinations of components are responsible for this effect [17]. In addition, although there are potential anti-prion agents or drugs based on nanoparticles or macromolecular structures such as dendrimers [18,19,20], polyoxometalates [21], humic acid [22], cellulose ethers [23], and protein-bound polysaccharide K [24], the potential of these agents as disinfectants and their inactivation mechanisms remain unclear. Taken together, a safe and effective disinfectant for inactivating prions is required.

Recently, we generated mesoscopic crystals, comprising a fine particle structure of ~50–500 nm, primarily composed of carbon and calcium from mineral components derived from plants [25]. By dissolving these mesoscopic crystals in water, an electrically charged disinfectant (CAC-717) was generated that can inactivate several viruses and bacteria [25,26,27]. Because calcium in the mesoscopic structure attracts protons from H_2_O, generating CAC-717 water with free OH^–^ ions, it is highly alkaline (pH~ 12.3). However, it becomes almost neutral on contact with human and animal tissue [25] and does not cause irritation [25]. Taken altogether, the above-mentioned effectiveness of CAC-717 as a disinfectant prompted us to further investigate CAC-717 as a potential disinfectant for prions.

Here we examined the ability of CAC-717 to inactivate prion proteins. Western blotting was used for PrP^Sc^ analysis, and protein misfolding cyclic amplification (PMCA), which mimics the in vivo reaction and amplifies PrP^Sc^ in vitro [28,29,30], was used to examine the seeding activity of PrP^Sc^. In both assays, PK-resistant PrP (PrPres) was used as an indicator of PrP^Sc^. Lastly, prion transmissibility was tested by a mouse bioassay. Our study shows that CAC-717 is a safe and useful disinfectant for scrapie prions.

## 2. Results

### 2.1. CAC-717 Decreases PrP^Sc^ in ScN2a Cell Lysates

**Figure 1:**
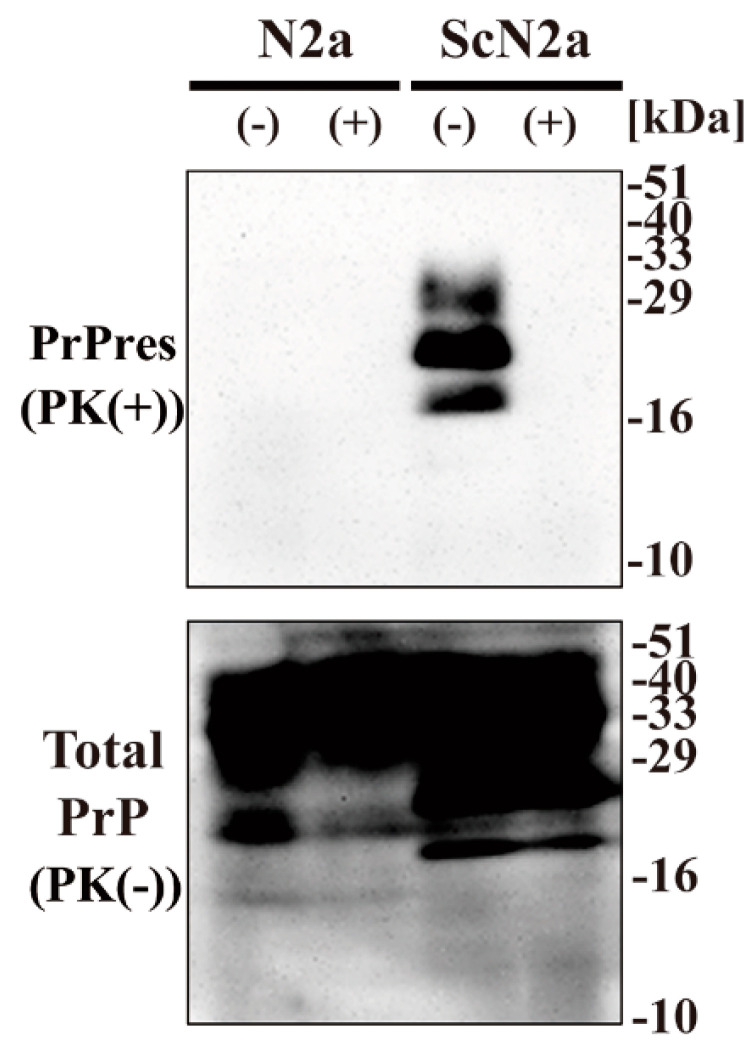
Reduction of Chandler scrapie prion in ScN2a cell lysate after CAC-717 treatment. Cell lysates of N2a cells (N2a) or scrapie prion (Chandler)-infected N2a cells (ScN2a) in phosphate-buffered saline (PBS) were mixed with an equal volume of CAC-717 (+) or PBS alone (–) as a negative control. Samples were incubated at 25 °C for 1 h and then subjected to either treatment with proteinase K (PK) to determine PrPres levels (PK(+)) or no treatment to determine total PrP (PK(-)). Samples were analyzed by SDS-PAGE followed by Western blotting using anti-PrP antibody SAF83. PrPres was not detected in the ScN2a cell lysate treated with CAC-717, but total PrP levels were almost the same in CAC-717-treated and PBS-treated samples of both uninfected N2a and infected ScN2a cells. Molecular mass markers (kDa) are indicated on the right-hand side of the gel.

To examine the effect of CAC-717 on prion proteins, we investigated whether CAC-717 treatment might reduce the amount of PrP^Sc^ detected in Western blotting. Cell lysate of mouse prion (Chandler)-infected N2a cells (ScN2a cells) or the uninfected counterpart (N2a cells) was mixed with an equal volume of CAC-717 (Figure 1). As a negative control, the samples were treated with phosphate-buffered saline (PBS). Mixtures were incubated at 25 °C for 1 h before determining the amount of prion protein (PrP) in each sample by Western blotting using anti-PrP antibody SAF83, which recognizes mouse amino acids residues 125–163 of PrP. As a result, PrPres was not detected in the ScN2a cell lysate treated with CAC-717; however, the amount of total PrP did not differ substantially between samples with and those without CAC-717 treatment (Figure 1).

### 2.2. Mouse Bioassays of CAC-717-treated ScN2a Cells

**Figure 2:**
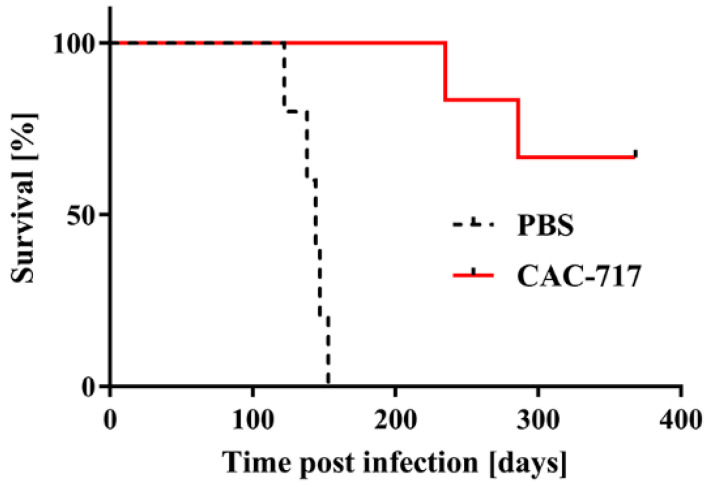
Mice injected with scrapie prions that had been pre-treated with CAC-717 survive longer than those injected with PBS-treated scrapie prions. Shown are the survival curves of Tga20 mice injected intracerebrally with CAC-717-treated Chandler prion-infected ScN2a cell lysate (red line) or the corresponding PBS-treated counterpart (dotted black line). Mice injected with CAC-717-treated prions survived significantly longer as compared with mice injected with PBS-treated prions (log-rank test, *p* < 0.01).

Next, we tested the infectivity of the CAC-717-treated cell lysate of ScN2a cells by intracerebral inoculation into Tga20 mice (Figure 2).

All five mice inoculated with PBS-treated ScN2a cell lysate succumbed to the disease within 140.8 ± 11.9 days; by contrast, only two of six mice inoculated with CAC-717-treated ScN2a cell succumbed to the disease within 235 and 286 days, respectively. The other four mice did not show any signs of scrapie up to the time of study termination (368 days post infection).

An accumulation of PrP^Sc^ in the brains of mice that succumbed to the disease was confirmed by immunohistochemistry, whereas no accumulation of PrP^Sc^ was detected in the brains of mice without signs of scrapie (Supplementary Figure S1). Western blotting for PrPres in the brains of the mice affected by disease also supported these findings (data not shown). Collectively, these results indicate that CAC-717 treatment decreases transmissibility of the scrapie prion.

### 2.3. Quantitative PMCA of CAC-717-Treated ScN2a Cells

**Figure 3:**
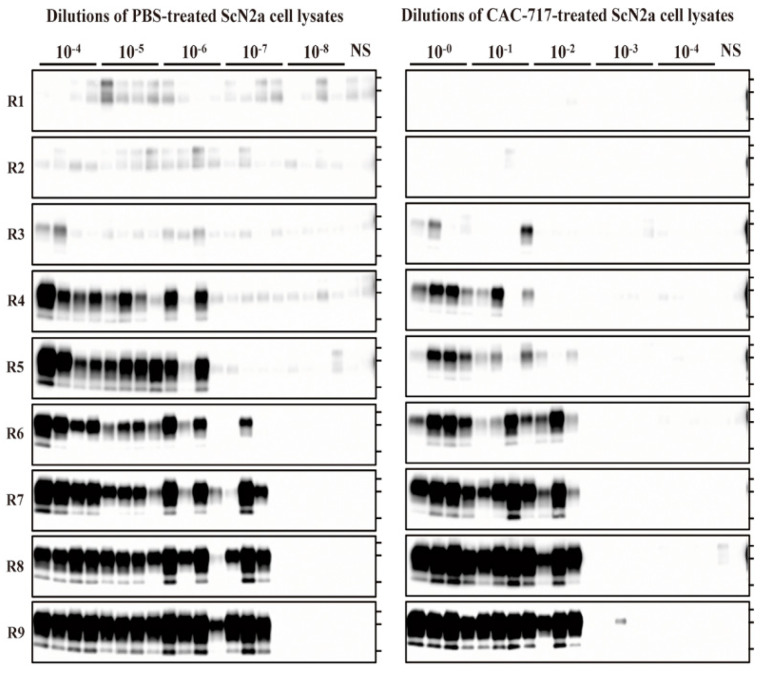
CAC-717 treatment reduces the amplification of PrPres examined by protein misfolding cyclic amplification (PMCA). Brain homogenate of CD-1 mice was used as the PrP^C^ substrate for PMCA. PBS-treated (left panel) or CAC-717-treated (right panel) ScN2a cell lysates were diluted 10^−4^ to 10^−8^ or 10^−0^ to 10^−4^, respectively, with PMCA buffer. Amplification was performed in quadruplicate except for the non-seeded control (NS), which was performed in duplicate. After PK digestion of the samples, PMCA products were analyzed by Western blotting using horseradish peroxidase (HRP)-conjugated T2 anti- PrP antibody.

To evaluate the seeding activity of ScN2a cell lysate treated with CAC-717 or PBS, quantitative PMCA analysis was applied. We performed end-point dilution PMCA on the ScN2a cell lysates that were used in the mouse bioassay and calculated the medium dose of PMCA (PMCA_50_). These cell lysates were serially diluted with PMCA buffer and subjected to nine rounds of PMCA (Figure 3).

For the PBS-treated samples, PrPres-positive signals were clearly detected in all quadruplicate samples until 10^−6^ dilution at round nine, and in three out of four samples at 10^−7^ dilution; however, all samples were negative at 10^−8^ dilution. For the CAC-717-treated samples, by contrast, PrPres signals were detected in all quadruplicate samples until 10^−1^ dilution, and in three out of four samples at 10^−2^; however, samples were almost negative at 10^−3^, and completely negative at 10^−4^ dilution. The Spearman–Käber estimate of the log PMCA_50_ per ml of PBS- and CAC-717-treated ScN2a cell lysate was 9.95 and 5.20, respectively, indicating a 4.75-log reduction. These observations suggest that CAC-717 treatment significantly reduced the seeding activity of PrP^Sc^.

## 3. Discussion

In this study, we examined the effect of CAC-717 on the inactivation of scrapie prions. In a preliminary screening, the effect of CAC-17 on lysates of prion-infected cells was examined by Western blotting. Whereas the CAC-717-treated cell lysate with no PK digestion showed PrP signals, the lysate treated with PK showed no PrP signals, indicating that PrP^Sc^ was not present in the lysate after CAC-717 treatment.

Next, we evaluated the conversion activity of any remaining PrP^Sc^ after CAC-717 treatment by using PMCA. A clear decrease in seeding activity of PrP^Sc^ was observed for samples after CAC-717 treatment. Furthermore, prion transmissibility was not observed until 230 days after treatment with CAC-717. These results may support the idea that CAC-717 leads to a reduction in PrP^Sc^ conversion activity and prion transmissibility.

Previous investigations have demonstrated that CAC-717 solution is useful as a disinfectant for many kinds of animal and human pathogens including influenza virus, human/mouse norovirus, feline calicivirus, and bacteria [25,26,27]. The energetic impact of the emitted pulsed waves is a nano-sized (50–500 nm) micro-environmental event that has no deleterious effects on higher multicellular organisms such as animals or plants. In addition, CAC-717 has been shown to be harmless and non-irritant to humans and animals because its pH reduces to 8.8 ± 1.2 immediately after application to the human body [25]. Similarly, after CAC-717 comes into contact with tissues or cells, the pH is neutralized. Therefore, although it is well-known that alkalinity potentiates the effect of proteolysis related to the inactivation of prions by an alkaline cleaner [31], we speculate that alkaline pH may not be a major contributor to the inactivation activity of CAC-717.

A previous study showed that freshly electrolyzed mineral water may also contain electrons and hydroxide ions, together with calcium hydrogen carbonate particles [25]. The reversible redox process observed in electrolytes containing calcium ions might be effective for electrolysis [25], which may lead to the inactivation of prions; however, the inactivation mechanisms of CAC-717 are not entirely understood. Overall, further studies on the mechanism by which CAC-717 inactivates prions are necessary.

The present study has demonstrated that prion transmissibility is significantly reduced after treatment by CAC-717; however, there are limitations to the results as follows. Although a 4.75-log reduction in seeding activity by CAC-717 was observed by PMCA, prion seeding activity does not always correlate with infectivity of prions [32]. As the reduction in prion titer due to CAC-717 in the bioassay was not determined in this study, the level of the reduction in prion titer by CAC-717 remains unclear. Therefore, further studies on prion titration using multiple dilutions of prion inocula, ideally derived from a brain homogenate, are required to quantify the reduction in prion infectivity titer. In addition, the effect of CAC-717 might vary among different prion strains, and/or animal species. Moreover, the nature of the treatment sample (e.g., brain homogenate, cell lysate, blood, or purified materials) might affect the efficiency of the inactivation of prions by CAC-717 treatment. For example, biomolecules such as proteins in the local environment around the prions may alter the inactivation efficiency [3]. Furthermore, because the present study was performed for a suspension/solution, the results cannot be extrapolated to surface decontamination. Therefore, further study is required to verify the efficacy of CAC-717 under various conditions. In addition, the reproducibility of the effects of CAC-717, including whether the anti-prion effect varies from batch to batch of CAC-717 (due to potential differences in production conditions or source materials such as plants, stones, fossil corals, and shells, etc.) or differs with pH, the amount of calcium hydrogen carbonate particles, or the different populations of carbon complex microparticles that might be present during manufacturing should be studied before practical use of this disinfectant. Clarification of the contact time required for prion inactivation and appropriate storage condition of CAC-717 is also required. Furthermore, although a previous study of CAC-717 on human norovirus (HuNV) found no significant difference in the inactivation of HuNV between CAC-717 and a commonly used disinfectant (sodium hypochlorite) [26], the relative effect of CAC-717 and other disinfections on prions remains unclear. Although CAC-717 does not cause irritation and is not corrosive, there are other non-toxic, non-corrosive, and effective prion disinfectants such as hypochlorous acid [33]. Therefore, a detailed comparison of CAC-717 with other prion disinfectants such as hypochlorous acid will be necessary in future studies.

In conclusion, our findings indicate that the electrically charged disinfectant CAC-717 is a potentially invaluable and safe disinfectant for prion inactivation. It is expected to be used as a disinfectant in the medical field for prion diseases.

## 4. Materials and Methods

### 4.1. Preparation of CAC-717

CAC-717 displays a pH of approximately 12.3 and contains 6.9 mM calcium hydrogen carbonate particles with a mesoscopic structure [25,26]. In accordance with Japan patent No. 5778328, CAC-717 (Food and Drug Administration/USA Regulation No. 880.6890 Class 1 disinfectant) was produced by mixing Solution (A) and Solution (B) in a 1:10 ratio. Solution (A) and Solution (B) were prepared as follows.

### 4.2. Preparation of Solution (A)

First, Material (A1) was prepared by mixing a 1:1 ratio of Material (A1-1) and Material (A1-2). Material (A1-1) comprised the dried grinds of Compositae plants, which were obtained by mixing 10% (*w*/*w*) field thistle (leaf part, stem part, and flower part), 60% (*w*/*w*) mugwort (leaf part and stem part), and 30% (*w*/*w*) *Farfugium japonicum* (leaf part and stem part), following by drying and pulverizing. Material (A1-2) composed family Rosaceae plants and was obtained by mixing 20% (*w*/*w*) *Rosa multiflora* (leaf part, flower part), 10% (*w*/*w*) *Geum japonicum* (leaf part and stem part), 70% (*w*/*w*) raspberry (leaf part, stem part, and flower part), followed by drying and pulverizing. Second, Material (A2) was prepared by mixing *Acer* (leaf part and stem part), *Betura platyphylla* var. *japonica* (leaf part, stem part, and bark part), *Cryptomeria japonica* (leaf part, stem part, and bark part) in proportions of 25% (*w*/*w*), 25% (*w*/*w*), and 50% (*w*/*w*), respectively, followed by drying.

Next, Material (A), obtained by mixing material (A1) and material (A2) at a ratio of 1:3, was added to distilled water at 12.5% (*w*/*v*) in apparatus described in Japan patent No. 5778328. Next, direct current (DC 8300 V, 100 mA) was applied to the conductive lines of the apparatus using a Teflon insulation-coated electrostatic field electrode (N-800N, Mineral Activation Technical Research Center, Kumamoto, Japan; Japan Patent No. 5864010). Simultaneously, water flow was generated around the conductive wires in the same direction as the direct current. During the electrification and water flow, ultrasonic vibration (oscillation frequency, 50 kHz; amplitude, 1.5/1000mm) was simultaneously applied to the water. Thereafter, the solution was exposed to far-infrared radiation at a wavelength of 6–14 μm, resulting in Solution (A).

### 4.3. Preparation of Solution (B)

Material (B1), Material (B2), Material (B3), Material (B4), Material (B5), and Material (B6) were prepared from various combinations of limestone, fossil coral, shell, and activated carbon. Material (B1) contains limestone, fossil coral, and shell at ratios of 70% (*w*/*w*), 15% (*w*/*w*), and 15% (*w*/*w*), respectively. Material (B2) is a mixture of limestone, fossil coral, shell, and activated carbon at 40% (*w*/*w*), 15% (*w*/*w*), 40% (*w*/*w*), and 5% (*w*/*w*), respectively. In Material (B3), limestone, fossil coral, and shell are mixed at 80% (*w*/*w*), 15% (*w*/*w*), and 5% (*w*/*w*), respectively. Material (B4) contains limestone, fossil coral, and shell at 90% (*w*/*w*), 5% (*w*/*w*), and 5% (*w*/*w*), respectively. Material (B5) comprises limestone, fossil coral, and shell at 80% (*w*/*w*), 10% (*w*/*w*), and 10% (*w*/*w*), respectively. Material (B6) contains limestone, fossil coral, and shell at 60% (*w*/*w*), 30% (*w*/*w*), and 10% (*w*/*w*), respectively. Solution (B) was produced by passing through distilled water through six types of vessels containing Material (B1), Material (B2), Material (B3), Material (B4), Material (B5), and Material (B6), respectively, as described in Japan patent No. 5778328.

### 4.4. Preparation of Cell Lysates

ScN2a I3/I5-9 cells [34], an N2a cell line infected by the scrapie Chandler isolate that persistently produces PrP^Sc^, and the corresponding uninfected N2a cells were used in this study. The cells were grown in OptiMEM (Invitrogen, Carlsbad, CA, USA) supplemented with 10% fetal calf serum (JRH Biosciences Inc., St Louis, MO, USA) and standard antibiotics (100 units/mL of penicillin and 100 µg/mL of streptomycin) at 37 °C in a humidified 5% CO_2_ atmosphere. Cell pellets of 80–90% confluent cells were collected after washing three times in PBS (Life Technologies, Carlsbad, CA, USA). A 10% (*w*/*v*) cell lysate was prepared by passage through a 28 G injection needle to obtain ScN2a cell lysate in PBS, followed by freeze-thawing at –80 °C.

### 4.5. CAC-717 Treatment

Cell lysates prepared as described above were mixed with an equal quantity of CAC-717 or PBS as a negative control. The mixture was incubated at 25 °C for 1 h. Samples were quickly frozen at –80 °C prior to analysis as described below.

### 4.6. Prion Inoculation

All animal experiments were performed according to the guidelines of the National Institute of Animal Health. All experimental procedures were approved by the Animal Ethics Committee of the National Institute of Animal Health (approval no. 17-011; approval date May 25, 2017) and due attention was paid to the welfare of the animals. To measure the change in infectivity after CAC-717 treatment, CAC-717-treated and PBS-treated ScN2a cell lysates were twice-diluted with PBS, and 20-µl aliquots were injected into the cerebral ventricle into Tga20 mice using a microsyringe. Clinical symptoms such as tremors and ataxia were observed at end-stage disease.

### 4.7. Western Blot Analysis

The protein concentration of samples was measured by using a Bio-Rad DC protein assay kit (Bio-Rad, Hercules, CA, USA). The sample (200 µg of protein per 100 µL) was incubated with or without PK at 20 µg/mL for 1 h at 37 °C to discriminate PrPres from PrP^C^ (only PrPres is detected after PK treatment, whereas total PrP are detected in the absence of PK treatment). An equal volume of 2× SDS gel-loading buffer (90 mM Tris/HCl (pH 6.8), 10% (*v*/*v*) 2-mercaptoethanol, 2% (*w*/*v*) SDS, 0.02% bromophenol blue, and 20% (*v*/*v*) glycerol) was added and the samples were heated at 100 °C for 10 min to terminate the reaction prior to Western blotting.

SDS-PAGE (12% gel) was performed to separate proteins as described previously [35,36]. The proteins were transferred to polyvinylidene difluoride membranes (Amersham Biosciences, Piscataway, NJ, USA) by using a semidry blotting system (Bio Rad, Cambridge, MA, USA). The membranes were then blocked with 5% skimmed milk (Wako, Osaka, Japan) for 1 h at room temperature, and further incubated for 1 h at room temperature with anti-PrP antibody SAF83 (SPI Bio, Montigny le Bretonneux, France) in PBS-Tween (0.1% Tween 20) containing 0.5% skimmed milk. After three washes in PBS-Tween for 10 min, the membrane was incubated with horseradish peroxidase (HRP)-labeled anti-mouse immunoglobulin secondary antibody (Jackson Immunoresearch, West Grove, PA) in PBS-Tween containing 0.5% skimmed milk for 1 h at room temperature, and then washed three more times in PBS-Tween for 10 min. An enhanced chemiluminescence reagent (Amersham Bioscience) was used to develop the blots and detect the chemiluminescence signal.

### 4.8. Protein Misfolding Cyclic Amplification

PrP^C^ substrates for PMCA were prepared from the brains of 6-week-old CD-1 mice. Brains were homogenized at 20% (*w*/*v*) in PBS containing proteinase inhibitor-cocktail (cOmpleteTM EDTA free, Roche Diagnostics, Mannheim, Germany), and then mixed with an equal volume of 2× PMCA buffer (2% Trition X-100 and 8 mM EDTA in PBS). After constant agitation at 4 °C for 1 h, digitonin (12333-51, Nacalai, Kyoto, Japan) and heparin (H3393, Sigma-Aldrich) were added to the brain homogenate at a final concentration of 500 μg/mL and 300 μg/mL, respectively. Aliquots of PrP^C^ substrate were stored at −80 °C until use. PMCA was performed by using an automatic cross-ultrasonic protein-activating instrument (ELESTEIN 070-GOT; Elekon Science Corp., Chiba, Japan) as reported previously [37,38] with slight modifications. For round one of PMCA, the ScN2a cell lysate that was used in the mouse bioassay was 10-fold serially diluted in PMCA buffer. Aliquots (2 μL) of CAC-717-treated ScN2a cell lysate dilutions from 1:10^0^ to 1:10^4^ or PBS-treated ScN2a cell lysate dilutions from 1:10^4^ to 1:10^8^ were added to 50 μL of PrP^C^ substrate in quadruplicate tubes containing a zirconia bead (YTZ-2 TOSOH, Tokyo Japan) and amplification supplement X (Morikazu Imamura, personal communication, March 6, 2019). The amplification method comprised 32 cycles of sonication (pulse oscillation for 3 s, repeated five times at intervals of 0.1 s), followed by incubation at 37 °C for 1 h with gentle agitation. For sequential rounds of PMCA, 10 μl of the amplified product obtained after the first round were diluted into 40 μL of PrP^C^ substrate and a new round of amplification was performed. This process was repeated for a total of 9 rounds. The PMCA products at each round were subjected to Western blotting for PrPres detection. The PMCA products were mixed with an equal volume of PMCA buffer containing 0.2% SDS and 80 µg/mL proteinase K, and incubated for 30 min at 37 °C. The digestion was stopped by adding lithium dodecyl sulfate sample loading buffer (Thermo Fisher Scientific). The samples were separated by 4–20% SDS-polyacrylamide gel electrophoresis, electroblotted onto PVDF membranes and probed with anti-PrP T2 antibody conjugated with horseradish peroxidase. The blots were developed with SuperSignal West Dura Extended Duration Substrate (Pierce, Rockford, IL, USA) and visualized by FluorChem (Alpha InnoTec, San Leandro, CA, USA). The medium dose of PMCA (PMCA_50_) was estimated from the results of the ninth round of quadruplicate amplification by using the 50% endpoint calculation method as previously described [39].

### 4.9. Statistical Analysis

Log-rank test was used to analyze differences in the survival curves of mice. Analysis was carried out by using GraphPad Prism 7 software version 7.02 (GraphPad Prism Software Inc., La Jolla, CA, USA).

## Supplementary Materials

The following are available online at https://www.mdpi.com/2076-0817/9/7/536/s1, Figure S1: Histopathological changes and PrP^Sc^ accumulation in the brain of mice that succumbed to disease.
