# Supplementary material for: Inactivation of Scrapie Prions by the Electrically Charged Disinfectant CAC-717
Source: Pathogens. 2020 Jul 3;9(7):536. doi: 10.3390/pathogens9070536 (PMC7400677; doi:10.3390/pathogens9070536)
Supplement: Supplementary file 1 [file pathogens-09-00536-s001.pdf]

Supplementary Materials

Supplementary File S1 Histopathological changes and PrP^{Sc} accumulation in the brain of mice that succumbed to disease.

Materials and Methods

Histopathology and Immunohistochemistry

Formalin-fixed left hemispheres of the brain were immersed in 98% formic acid to reduce infectivity and embedded in paraffin wax. Serial sections were stained with hematoxylin and eosin (H&E) to evaluate neuropathological changes. After epitope retrieval by autoclaving in 0.01 M citrate buffer (pH 6.0), immunocytochemistry of PrP^{Sc} was performed with monoclonal anti-PrP antibody 2G11. Immunoreactions were developed by using anti-mouse universal immunoperoxidase polymer (Nichirei Histofine Simple Stain MAX-PO (M); Nichirei, Tokyo, Japan) as the secondary antibody, and visualized with 3,3'-diaminobenzedine tetrachloride as the chromogen.

Results

Accumulation of PrP^{Sc} in the Brains of Mice that Succumbed to Disease

Histopathological analysis showed the presence of mild to moderate spongiform changes in the grey matter of the brain stem and cerebellar nuclei of all mice that succumbed to disease (all 5 mice injected with PBS-treated scrapie prion, and 2 of 6 mice injected with CAC-717-treated scrapie prion). Immunohistochemistry with antibody 2G11 showed that PrP^{Sc} accumulated in the cerebral cortex, grey matter of the brain stem, and cerebellar nuclei of all succumbed mice (again, all 5 mice injected with PBS-treated scrapie prion, and 2 of 6 mice injected with CAC-717-treated scrapie prion), whereas no such accumulation of PrP^{Sc} was seen in mice that survived (4 of 6 mice injected with CAC-717-treated scrapie prion).

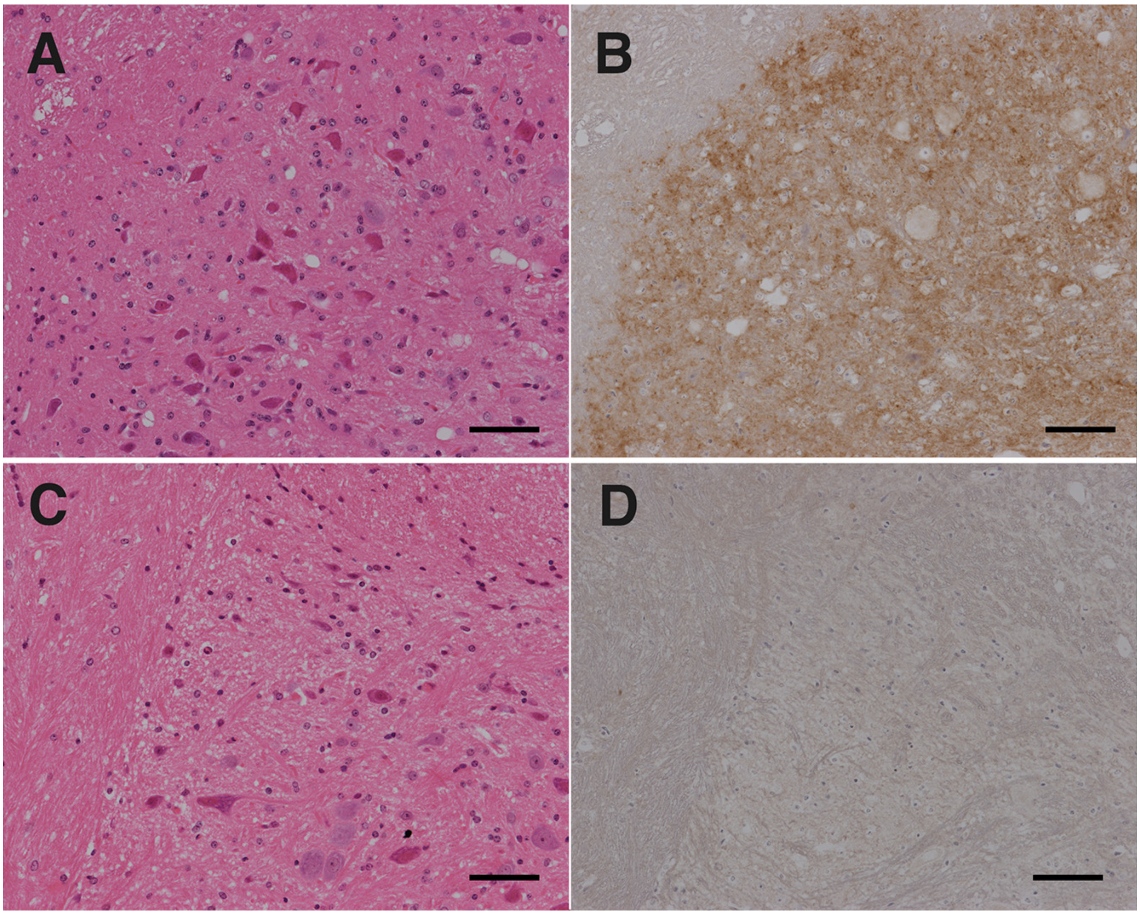

Figure S1 Histopathological changes and PrP^{Sc} accumulation in the brain of mice. (**A**) H&E staining showed mild spongiform changes in the cerebellar dentate nucleus of a succumbed mouse inoculated with PBS-treated scrapie prions. (B) Accumulation of PrP^{Sc} was confirmed by immunohistochemistry in the nucleus of a succumbed mouse inoculated with PBS-treated scrapie prions. (C) H&E staining showed no change in the cerebellar dentate nucleus of a surviving mouse inoculated with CAC-717-treated scrapie prions. (D) Immunohistochemistry showed no PrP^{Sc} accumulation in the nucleus of a surviving mouse inoculated with CAC-717-treated scrapie prions. Bars, 50 μm.
